# Music therapy is a potential intervention for cognition of Alzheimer’s Disease: a mini-review

**DOI:** 10.1186/s40035-017-0073-9

**Published:** 2017-01-25

**Authors:** Rong Fang, Shengxuan Ye, Jiangtao Huangfu, David P. Calimag

**Affiliations:** 1 Department of Medicine, Section of Neurology, Saint Anthony Hospital, 2875 West 19th Street, Chicago, IL 60623 USA; 2 Intent Inc., Ningbo, Zhejiang 315000 China; 3 Laboratory of Applied Research on Electromagnetics (ARE), Zhejiang University, Hangzhou, 310027 China

**Keywords:** Alzheimer’s disease (AD), Music therapy (MT), Cognition impairment, Non-pharmacological intervention, Clinical trial

## Abstract

Alzheimer’s Disease (AD) is a global health issue given the increasing prevalence rate and the limitations of drug effects. As a consequent, non-pharmacological interventions are of importance. Music therapy (MT) is a non-pharmacological way with a long history of use and a fine usability for dementia patients. In this review, we will summarize different techniques, diverse clinical trials, and the mechanisms of MT as it is helpful to the cognition in AD, providing reference for future research. Many articles have demonstrated that MT can reduce cognitive decline especially in autobiographical and episodic memories, psychomotor speed, executive function domains, and global cognition. MT is a promising intervention for strategy of dementia especially of AD and it must be started as early as possible. However, more evidences with prospective, randomized, blinded, uniform and rigorous methodological investigations are needed. And we should consider to combine MT with other cognitive stimulations such as dance, physical exercise, video game, art and so on.

## Background

Alzheimer’s Disease (AD), which is the most common type of dementia, is a neurodegenerative disease characterized by progressive cognitive impairment and neuropsychiatric symptoms [1, 2]. It can cause patients to lose their daily living abilities. The pathological features of AD are accumulated amyloid beta (Aβ) protein in senile plaques and tau protein in neurofibrillary tangles, as well as the loss of neuron and synapse [3]. AD is one of the greatest health challenges of this century for humanity. With the increasing aging population, the numbers of AD and other dementias are growing dramatically. There is a report which says the number of AD patients has reached over 35 million worldwide in 2013, and this number is estimated to triple by 2050 [4]. However, there is lack of effective disease-modifying drugs for AD [5, 6].

Considering the growing population of AD and the severe harmfulness to patients’ health, non-pharmacological treatments play a more and more important role in preventing and relieving symptoms of AD, such as physical exercise, music therapy (MT), diet and so on [7, 8].

MT is an important method for neuropsychological, cognitive, and social behavior goals in the field of dementia with low-cost [9]. It requires that research, practice, education, and clinical training are based on professional standards in MT (World Federation of Music Therapy (WFMT)) [10]. Many researches have demonstrated that MT is beneficial to improve cognition and to reduce neuropsychiatric syndromes of AD [10, 11]. Since the absence of side effects and the convenience to operate for AD patients and their caregivers, MT becomes an ideal option for intervention. In this review, we will refer to recent articles to summarize different techniques, diverse clinical trials, and the mechanisms of MT in impacting cognition in AD patients, thus providing reference for future research.

## Evidence and method

We have planned and analyzed literatures starting from reviews and systematic reviews, concerning AD and music, for recent 5 years in peer-reviewed journals. Than we have searched PubMed database for this review. The language was restricted to English and Spanish. And the publication range was from January 2006 to October 2016. We used keywords ‘Alzheimer’s disease/AD/dementia of the Alzheimer’s type/Alzheimer dementia/dementia’ in combination with ‘music/music therapy/music listening/singing’. The included criteria were as follows for this review: (1) randomized trials or observational studies (including cohort and case-control studies), as well as reports, letters, reviews, or conference abstracts; (2) patients were diagnosed with dementia using clinically diagnostic criteria; (3) the result was on the aspect of relationship between music therapy and dementia. Information especially about cognitive effects on AD patients was retrieved from these articles included: the first author, publication year, sample size, music therapy technique, therapy time, and mainly results (see Table 1). An overview on cognitive effects of music therapy in AD patients is provided.

## Different techniques of MT for AD

Music has been reportedly used in the field of dementia for many years [12]. And many different techniques with the sound elements can be observed in different clinical trials and studies. MT is always conducted by a qualified music therapist [10], so that the therapist can mediate the music treatment according to different patients based on the psychological and/or rehabilitative approaches [9]. Because of the wide and heterogeneous range of applications, there might be some direct influence on the results of MT for dementia. So we will summarize different techniques of MT for dementia especially for AD in this paragraph, including listening to the music, singing songs, music-based intervention, background music, music with activities and multisensory stimulation.

### Listening to the music

There are many researches using receptive music for listening in the MT [11]. Johnson JK reported that the AD twin had a significant improvement on the spatial-temporal task after listening to a piece of piano sonata of Mozart in 1998 [13]. Last year Li CH et al. used the method of listening to Mozart’s Sonata (KV 448) and Pachelbel’s Canon with headphones for mild AD patients. Results showed that the scores of Cognitive Abilities Screening Instrument (CASI)-estimated Mini-Mental State Evaluation (MMSE) and CASI were less decreased after 6-month music intervention than control group but without statistical significance, and the cognition of abstraction domain was better in MT group [11]. Other documents verified that a particular kind of music could also mediate the effect of the music, such as familiarity or preference for patients [14]. A study conducted by Arroyo-Anlló EM et al. demonstrated that listening to the familiar Spanish songs showed a stabilization or improvement in self-consciousness (SC) of AD in mild or moderate stage. And the AD group with familiar songs performed better in MMSE and Frontal Assessment Short (FAS) tests than the group with unfamiliar songs [15].

### Singing songs

Singing is also widely used for dementia. Satoh M et al. made 10 AD patients sing their favorite songs for 6 months using karaoke [10]. Karaoke is a method with music automatically played accompaniment when patients are singing. Many people are very familiar with karaoke, which is used universal and is enjoyable. People can mediate their own voice during the accompaniment [16]. Results showed time for Japanese Raven’s Colored Progressive Matrices reduced and the neuropsychiatric symptoms improved after 6-month MT [10]. In addition to the above, Meilán García JJ et al. compared different kinds of emotional music (including happy, sad, cafeteria sound, music without an emotional component, and absence of sound) as MT separately. They found that music with sad emotion was the most effective for the recall of autobiographic experiences especially for the remote memories [17]. Which indicated that the emotion in the music played an important role in the recall memory process of dementia.

### Music-based intervention

This technique always calls for the music therapist using music elements like rhythm or melody as accompaniments to help patients to remember verbal contents. Simmons-Stern NR et al. first compared the recognition effectiveness of sung lyrics and spoken lyrics in AD patients in 2010. They found that music could enhance the brain encoding capacity of verbal information compared with spoken [18]. Moreover, an interesting study conducted by Palisson J and his colleagues, who compared the verbal text mnemonic effects with three different accompaniments (including sung by the Ode to Joy by Beethoven, spoken by Modern Times by Charlie Chaplin (movie sequence), or spoken alone). The data showed that sung texts were better remembered than other two groups [19].

### Background music

There are rare clinical documents using music as background in dementia patients. Vivaldi’s ‘Spring’ movement from ‘The Four Seasons’ was once used as a background music during the recall tests in mild AD patients and healthy controls. Results indicated that music background could enhance the recall effects of autobiographical memory and reduce anxiety emotions [20]. However, the mechanism of the effect of background music remains controversial. Irish M et al. thought the change of mood such as anxiety reduction was the reason [20]. Some scientists considered the arousal heightened by music could improve memory scores [21].

### Music with activities

There are more and more researches using MT which comprises not only just music but also with other activities like singing, dancing, playing instruments, rhythmic movements and so on [22–25]. Särkämö T et al. separated patients with mild and moderate dementia into three groups (including singing with rhythmic movements group, listening with reminiscence and discussion group, and control group). The emotions and cognitions were improved in both singing and listening groups after 10-week intervention [23]. Gómez Gallego M and his workmates asked 42 mild to moderate AD to not just listen to the music they like, but also to greet, dance, play instruments and so on. The result of 6-week intervention indicated that music with other activities could improve cognitive status and alleviate neuropsychiatric symptoms of AD patients at the same time [22].

### Multisensory stimulation

In addition to pharmacotherapy for dementia, there is a tendency of increasing researches using the intervention combining several different cognitive stimulations, which is called multisensory stimulation. Ozdemir L and his colleagues synthesized multisensory stimulations including MT using instrument with a light tempo, painting inanimate animate pictures, and orientation to time-place-person as an intervention for mild AD patients. This research demonstrated that multi-domain stimulation could improved MMSE scores and decreased the scores of Geriatric Depression Scale and Beck Anxiety Scale [26]. A recent article has reported that the 6-month multisensory cognitive stimulation including art, music, exercise, recollection and horticultural therapy improved the memory test scores and the domain of community affairs of AD patients [27]. Moreover, Boulay M et al. demonstrated that MINWii, which was a music therapy video game, was indeed usable by AD patients. This video game comprised music therapy, physical exercise, and other cognitive stimulations. Dementia patients felt very satisfied with it [28]. Ben-Sadoun G and his workmates verified that Serious exerGames (SeG) which was also a video game was adaptive for neurodegenerative diseases including AD [29]. Next step is to explore the cognitive training effect of the video game for dementia patients and the result is worth expecting.

## The potential effect of MT for improving cognition of AD

Although most articles have indicated that MT has helpful effects for AD, the opinions on the effect of cognition are inconsistent [11]. There are many articles which have found that MT can reduce the mood symptoms and behavior disorders in dementias, especially depression, anxiety and agitation [30–33]. Yet the number of researches on the cognition topic of MT for dementia is lesser. Recently, scientists have paid more attention on the cognitive effect in AD patients with MT (see Table 1).

Increasing articles have demonstrated that MT can improve multiple domains of cognitions in AD patients, including attention, psychomotor speed, memory, orientation and executive functions [10, 23, 25, 26]. Bruer RA and other scientists found that listening to the music could increase the global cognition of AD [25, 34]. And Ozdemir L pointed out the effect of MT for AD could last for at least 3 weeks after intervention [26]. After 6-week intervention, Gómez Gallego M et al. found that listening to the music which patients like could significantly improve the memory and orientation of AD. At the same time, improvements were observed in depression and anxiety in AD patients. In addition, anxiety was reduced in mild ones, and delirium, hallucinations, agitation, irritability, and language disorders were reduced in moderate AD ones [22]. Kim HJ et al. demonstrated that multi-domain cognitive stimulation including music therapy could improve the word-list recognition and recall test scores. Besides, the domain of community affairs of AD patients and the Quality of Life (QOL)-AD of caregivers were also better [27]. Satoh M et al. indicated that listening and singing could improve psychomotor speed in AD after 6-month treatment. Decreasing Neuropsychiatric Inventory (NPI) score and prolonging sleep time were also observed after MT [10]. The clinical trial conducted by Arroyo-Anlló EM et al. showed that listening to unfamiliar music decreased the scores of MMSE and FAS, whereas the cognitive test scores did not vary in the familiar music group [15]. Which suggest the preventive and protective effects of music therapy in AD process. However, trials above were almost about dementia patients at the mild or moderate stage. AD patients at severe stage might not cooperate with scientists to complete MT trials because of physical or cognitive problems. For example, they might not usually sing [25], or could not take part in the neuropsychological tests, and so on. So there were few studies about the severe AD patients. Narme P et al. compared MT with cooking therapy in moderate or severe AD or mixed dementia patients. Result showed that both music and cooking improved patients’ emotions and ameliorated their behavioral disorders, yet no benefit was on the cognitive status [24].

In addition, Simmons-Stern NR et al. found that compared with spoken lyrics, AD and healthy older adults showed better scores on the memory test of sung general lyric content. However, participants in two groups performed equally on memory tests of specific lyric content both by sung and spoken. This indicated that music could enhance the preferential sensibility to a familiarity-based content rather than increase the recollection of memory process [35]. Palisson J et al. also verified that sung texts were better remembered than spoken texts for both AD and healthy groups, which indicated music during the encoding stage could facilitate learning and retention [19]. Nevertheless, another research conducted by Simmons-Stern NR et al. in 2010 showed that just AD patients had better recognition accuracy for the sung lyrics than the spoken ones, which did not exist in healthy older adults group [18]. So scientists proposed that music allowed a more holistic encoding to facilitate recognition only in AD patients, and music heightened arousal through better attention in AD patients [18].

**Table 1:** Clinical trials of MT for the cognition of AD

Reference	Music therapy technique	Sample	Therapy time	Mainly results
Irish M et al., 2006 [20]	Vivaldi’s ‘Spring’ from ‘The Four Seasons’ on a cassette recorder as a background during the test	10 mild AD, 10 healthy elderly; everyone is in MT and silence condition	two occasions (silence and music) at the same time of day, exactly 1 week apart	The recall on the autobiographical memory of AD in music condition improved; A significant reduction was on the State Trait Anxiety Inventory in the music condition.
Bruer RA et al., 2007 [25]	listen to the songs including singing along and playing instrument	17 dementia with cross-over design (see video in control condition)	1 h/week × 8 weeks	The scores of MMSE improved immediately after and the next day of MT compared with control group.
Ozdemir L et al., 2009 [26]	multisensory stimulation (including MT, painting pictures, and orientation interventions)	27 mild AD	4 sessions/week × 3 weeks	The scores of MMSE increased, and the scores of Geriatric Depression Scale and Beck Anxiety Scale decreased. This effect continued for three weeks after completion.
Meilán García JJ et al., 2012 [17]	different five kinds of music, including happy, sad, cafeteria sound, music without an emotional component, and absence of sound	25 AD (with five sessions individually)	30 min/session, and each session spaced by at least one week	Sad music was found to be the most effective to autobiographic memory.
Arroyo-Anlló EM et al., 2013 [15]	listen to the Spanish songs	20 AD with familiar music, 20 AD with unfamiliar music; all were mild or moderate AD	2–4 min/session × 3 sessions/week × 3 months	AD patients who received a familiar music intervention showed a stabilization or improvement in aspect of SC. The AD group with unfamiliar music showed poorer scores in MMSE and FAS test after intervention, whereas the familiar music group did not vary in their cognitive performance.
Narme P et al., 2014 [24]	music played on a CD player, and excerpts covered different styles (e.g., classical instrumental, familiar songs); Participants were asked to participate by singing and/or by using percussion instruments	moderate or severe AD, 18 in MT group, 19 in cooking group	1 h × twice a week × 1 month	Both music and cooking improved the patients’ emotional state and decreased the severity of their behavioral disorders, as well as reduced caregiver distress. No benefit was on the cognitive status.
Särkämö T et al., 2014 [23]	sing/listen familiar songs with vocal exercises; rhythmic movements (singing group) and reminiscence and discussions (listening group)	mild and moderate dementia: 27 in singing group, 29 in listening group, 28 in control group	1.5 h/session, weekly to daily × 10 weeks	Both singing and music listening improved mood, orientation, and remote episodic memory to a lesser extent, also attention and executive function and general cognition. Singing enhanced short-term and working memory and caregiver well-being, whereas music listening had a positive effect on QOL.
Satoh M et al., 2015 [10]	Singing training (using karaoke and the YUBA Method, passive listen and sing themselves)	AD: 10 in MT group, 10 in control group	once a week (60 min) or more × 6 months	Time for Japanese Raven’s Colored Progressive Matrices reduced. NPI score decreased. Sleep time prolonged.
Li CH et al., 2015 [11]	listen to Mozart’s Sonata (KV 448) and Pachelbel’s Canon with headphones	Mild AD: 20 in MT group, 21 in control group	30 min daily in the morning and before sleep × 6 months	CASI-estimated MMSE and CASI in the MT group were less decreased than control group without statistical significance, and the change of abstraction domain in the MT group was better. The score changes of NPI had no statistical significance in two groups.
Palisson J et al., 2015 [19]	Three texts were separately sung by the Ode to Joy by Beethoven (melody), spoken/recorded by Modern Times by Charlie Chaplin (movie sequence), or spoken alone; each text was visually presented.	12 mild AD, 15 healthy controls; either a musical (sung) or a nonmusical association (spoken associated to a silent movie sequence) or without association (spoken alone)	Each text contained eight lines, and texts were ask individual to remember them step by step.	Sung texts were better remembered than spoken texts for both groups, both immediately and after a retention delay.
Kim HJ et al., 2016 [27]	multidomain cognitive stimulation (art, music, recollection and horticultural therapy); MT involved playing melodies and/or accompanying chords for popular songs	AD with CDR = 1; 32 training, 21 controls	1 h × five times/week (MT just for once a week) × 6 months	Training group showed improvement in the word-list recognition and recall test scores versus the control. No change in the overall CDR score, but the domain of community affairs improved in the cognitive intervention group. QOL-AD of caregivers was slightly improved in the intervention group.
Gómez Gallego M et al., 2016 [22]	listen to the music which they like; patients should greet, dance, play instruments and so on	42 mild to moderate AD	45 min/session, twice a week × 6 weeks	Significant improvement was observed in memory, orientation, depression and anxiety in AD patients. In addition, improvement was observed in anxiety in mild ones, and in delirium, hallucinations, agitation, irritability, and language disorders in moderate AD patients.

*MT* music therapy, *AD* Alzheimer’s Disease, *MMSE* mini-mental state evaluation, *SC* self-consciousness, *FAS* frontal assessment short, *QOL* quality of life, *NPI* neuropsychiatric inventory, *CASI* cognitive abilities screening instrument, *CDR* clinical dementia rating

## The mechanism of MT for AD

Though there are increasing articles on the cognitive effect of music therapy for AD, the mechanisms of music therapy are less discussed. Neuroplasticity mechanism, neurogenesis, regeneration and repair mechanism, neuroendocrine mechanism and neuropsychiatric mechanism are the four main theories.

### The neuroplasticity mechanism

Satoh M et al. used functional Magnetic Resonance Imaging (fMRI) to detect the change of the brain function while AD patients sang familiar songs with a karaoke device. Result showed that the time for completing Japanese Raven’s Colored Progressive Matrices reduced after 6-month music training for AD compared with control group. And increased neural activities in the right angular gyrus and the left lingual gyrus in the before-minus-after subtraction analysis were observed in the MT group by fMRI technique [10]. Which indicated that MT with singing training could improve the neural efficacy of cognition in AD patients. Which also reflected that music might play an important role in the neuroplasticity mechanism in AD brain [34].

### The neurogenesis, regeneration and repair mechanism

There is one study showed that music influenced cranial nerves from fetus to adult in humans. Scientists have found that music had an effect on neuronal response and changed the cell counts [36]. Besides, Särkämö T et al. carried out a clinical research which showed music listening could promote neurons recovery and cognitive reservation during the early post-stroke stage [37]. Because there were evidences that steroids regulated neurogenesis, neuroprotection and cognition [38], and a strong relationship between music activity and steroid hormones was found [39, 40]. As a result, Fukui H et al. proposed that the neurogenesis, regeneration and repair of neurons by listening to music was one of the mechanisms through adjusting the level of steroid hormones [38].

### The neuroendocrine mechanism

Past articles have demonstrated that music therapy had an influence on levels of hormones including cortisol (C), testosterone (T) and estrogen (E) [38]. Moreover, Fukui H et al. recruited patients with AD to listen to chosen music and songs with verbal contact from the music therapist. After 1-month intervention, the problematic behaviors such as poriomania (fugue) decreased with a significant secretion of 17β-estradiol and testosterone [41]. Which meant that hormones had preventive influence on patients with AD through music therapy. Considering that hormones (i.e., 17β-estradiol) help to prevent exacerbation of AD, but have severe untoward side effects, MT is a better choice for hormone replacement therapy because it is noninvasive and safe [38].

At the same time, some studies suggested that music promoted several neurotransmitters, neuropeptides and other biochemical mediators to release, such as endorphins, endocannabinoids, dopamine and nitric oxide [42]. Which implied that music took part in the reward, stress and arousal, immunity, and social affiliation systems of the human beings [43].

### The neuropsychiatric mechanism

There is another opinion that emotions influence the cognitive test scores of AD patients rather than music therapy. Interestingly, almost all the researches listed in Table 1 have suggested that MT has the treatment effects on neuropsychiatric symptoms along with the cognition efficacy [22]. Irish M et al. used Vivaldi’s ‘Spring’ from ‘The Four Seasons’ as a background to verify that recall on the autobiographical memory of AD in music condition improved. And the anxiety decreased meanwhile, which reflected anxiety reduction might be one of the mechanisms for enhancing autobiographical memory recall with music [20]. As a supplement, Meilán García JJ et al. found that sad music was the most effective to autobiographic memory. So he pointed that music itself could not evoke memory. Instead, the neuropsychiatric symptom associated with music had a great effect on semantic memory [17].

## The prospect of MT for AD

Although there are some researches which demonstrate that MT is beneficial for preserving cognition of dementia especially of AD, these are not convincing enough. So the evidence of its effectiveness is still insufficient. And we need much more clinical trials with cohort, randomized, blinded, uniform (such as uniformities of frequency, time of intervention, and different kinds of control), and rigorous methodological investigations of the MT not only just for the immediate effect, but also for the long-time effect [44]. Another important thing which we must note is that MT is just a complementary therapy in the strategy for AD. Most researchers did not stop anti-dementia drug interventions especially Acetylcholinesterase inhibitors during MT [10, 11, 15, 23, 24]. And neuroscientists always did not change the doses of anti-dementia medications with MT during the clinical trials. Besides, many evidences are coming from dementia patients at the mild and moderate stage [11, 19, 22, 23, 26, 27]. So we should not stop drug interventions during MT and MT must be started up as early as possible. It is likely that the effect of MT for protecting cognition is not significant when patients are at the severe stage.

In addition to above, combining MT with other adjuvant interventions such as dance, art, video game, physical exercise, and so on, is another area for research and clinical use [28]. The unified method may improve motor and cognitive impairment, in company with reducing psychiatric symptoms of AD patients. And the synergistic action between MT and the therapists for the improvement effect of AD is raised. A document pointed out that decreased problematic behaviors of AD were found with MT and verbal contact from the therapist [41]. So a professional and excellent therapist also plays an important role during the course of MT.

## Conclusions

MT can be considered a non-pharmacological intervention which has the potential effects to reduce cognitive decline, improve neuropsychiatric symptoms, and enhance the QOL of AD [34]. Researches have demonstrated that MT can protect cognition of AD especially autobiographical and episodic memories, psychomotor speed, executive function, and global cognition. However, it is just an adjunct method for interventions of AD. So patients should not discontinue medications during MT and it must be started at the early stage of dementia even before dementia. Besides, more clinical trials with prospective, randomized, blinded, uniform and rigorous methodological investigations are needed to add more evidences to support the effect of MT for AD. And combination method with dance, art, video game, physical exercise, and so on is excitingly helpful. We should make a therapeutic strategy individually according to preference and physical endurance of every patient.

## Abbreviations

AD: Alzheimer’s disease
C: Cortisol
CASI: Cognitive abilities screening instrument
CDR: Clinical dementia rating
E: Estrogen
FAS: Frontal assessment short
fMRI: functional magnetic resonance imaging
MMSE: Mini-mental state evaluation
MT: Music therapy
NPI: Neuropsychiatric inventory
QOL: Quality of life
SC: Self-consciousness
SeG: Serious exerGames
T: Testosterone
WFMT: World Federation of Music Therapy
